# Management and Biosecurity Practices of Pig Farms in Nepal

**DOI:** 10.1002/vms3.70839

**Published:** 2026-03-09

**Authors:** Sachin Shrestha, Alok Dhakal, Ramjee Ghimire, Min Bahadur Oli, Rakesh Kumar Yadav, Nishant Shah

**Affiliations:** ^1^ Paklihawa Campus Institute of Agriculture and Animal Science, Tribhuvan University, Rupandehi Bhairahawa Nepal; ^2^ Michigan State University East Lansing Michigan USA

**Keywords:** African swine fever, Nepal, Pig farm

## Abstract

The swine industry is vulnerable to different infectious diseases. The pig industry and pork consumption have increased in Nepal for the past several years, but African swine fever in 2022 affected this industry badly. One of the possible ways to prevent disease is by employing good hygiene and strict biosecurity measures in farms. This study, conducted in 2023 in Dang and Jhapa districts of Nepal, sought to assess the management and the biosecurity practices across the pig farms. A survey conducted among pig growers included questions on major management practices, biosecurity measures (isolation, traffic control, sanitation) and demographics of respondents. Farm owners indicating having good practices in place as related to each question were given a score of one and zero to those indicating not having good practices in place. The findings showed the cumulative scores for all three biosecurity components to be 9.1 ± 3.0 (mean ± SD). The farmers lacked basic knowledge on biosecurity measures to be implemented in the farm. The data and analysis lead to the conclusion that there is a strong need for appropriate policies and programmes for helping and motivating pig farmers to adopt biosecurity practices and reduce the disease risk in Nepal.

## Graphical abstract

1) Biosecurity adoption level of pig farmers in Nepal is low.

2) The pig farms are highly vulnerable to infectious diseases like African swine fever.

3) Governments of Nepal should take immediate actions focusing on capacity building of pig farmers, information dissemination and adoption of biosecurity practices.

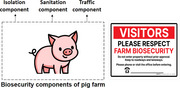

## 1 Introduction

The pig industry in Nepal has experienced steady growth over the past decade, driven by rising pork demand and its role as a critical source of livelihood for marginalized communities. As of 2023, the estimated pig population in Nepal was 1.35 million raised by 0.42 million holdings (MOALD 2024). The majority of farmers in Nepal raise pigs in scavenging systems and backyard farming, with only a few farms raising pigs in semi‐intensive and intensive systems (Pant 2017). The pig farming remains vulnerable to devastating disease outbreaks. In March 2022, the first reported case of African swine fever (ASF) in Kathmandu triggered a nationwide crisis (Subedi et al. 2022). Despite receiving early warnings for the ASF incursion in Nepal (Acharya and Wilson 2020), necessary programmes to prevent, detect and respond outbreaks were not yet tailored to the farmer's level and beyond. Due to the lack of proper quarantine and biosecurity measures, pig farmers suffered substantial economic losses resulting from the high mortality and morbidity associated with ASF. Report indicates that more than 17,000 pigs have died of ASF in Nepal since its incursion in 2022 (WOAH 2024), with many more cases going unreported. This led to the decline in pig population in Nepal in 2023 by 9.7% compared to the previous year (MOALD 2024).

Implementation of biosecurity practices is one of the safest methods of preventing emerging and re‐emerging diseases from entering livestock farms (Bottoms et al. 2013). Biosecurity refers to a set of management practices designed to prevent the introduction, spread and persistence of pathogens within and between animal populations (Food and Agriculture Organization 2010). With no commercial vaccine available for ASF and high cost for routine swine vaccination, biosecurity practices that farmers can implement are a viable defence against swine diseases (Bremang et al. 2022). Employing a high‐level of biosecurity in farms helps ensure animal welfare, reduces antimicrobial usage and improves production efficiency (Raasch et al. 2018; Stygar et al. 2020). On the contrary, the lack of good biosecurity measures may lead to the outbreak of several infectious diseases, including but not limited to foot‐and‐mouth disease (FMD), classical swine fever (CSF), Aujeszky's disease, porcine respiratory and reproductive syndrome (PRRS) and porcine epidemic diarrhoea (PED) (Elbers et al. 2001; Amass et al. 2004; Olugasa and Ijagbone 2007; Ellis‐Iversen et al. 2011; Lowe et al. 2014; Bremang et al. 2022). Farm biosecurity level is determined by its practices in isolation, traffic control, and sanitation (Cardona and Kuney 2002). Isolation refers to activities that limit contact with infectious agents within and around farm. Traffic control refers to controlling movement of personnel and equipment in and out of the farm to limit disease spread. Sanitation means keeping farms clean and healthy by regularly cleaning and maintaining hygiene in the farm surrounding, feeding area, watering area, and farm equipment. Farms can attain a high‐level of biosecurity if they strictly follow isolation, traffic control and sanitation procedures.

Several studies across diverse geographical regions have highlighted significant challenges in biosecurity practices on pig farms, indicating a global need for improvement. Singh et al. (2023) documented that farms in India exhibited poor biosecurity practices, including swill feeding, low adoption of fencing and footbaths and generally weak adherence to standard protocols, while Viltrop et al. (2021) reported that farms affected by ASF in Estonia exhibited markedly lower biosecurity levels compared to unaffected counterparts. Similarly, Jori et al. (2022) found that in France, frequent outbreaks of oedema disease were associated with farms that had insufficient isolation and traffic control measures, thereby increasing interactions with wild boars. In Poland, Dors et al. (2018) observed improved biosecurity compliance following the ASF outbreak, although critical gaps in fencing remained a persistent risk of disease introduction through human or wild boar interactions. Alarcón et al. (2019) reported that pig farms in Argentina had weak traffic control and isolation practices, and Kouam et al. (2020) revealed that pig farms in Cameroon performed poorly in all three biosecurity components, namely isolation, traffic control and sanitation, rendering them particularly susceptible to infectious diseases. Collectively, these findings suggests that although disease outbreaks have, in some regions, catalysed improvements, many pig farms worldwide remain vulnerable due to systemic biosecurity lapses, indicating the need for further quantitative analyses, policy‐oriented evaluations and comparative studies.

Understanding the biosecurity levels of pig farms is crucial for preparing the industry for emerging diseases, particularly for assessing risks and implementing effective outbreak control measures (Sahlström et al. 2014). Within these contexts, this study aimed to assess management and biosecurity practices adopted by pig farmers in Jhapa and Dang districts in Nepal. As per our knowledge, to date, no published studies have systematically assessed biosecurity and management practices in Nepal's pig farming sector, leaving a critical gap in understanding disease risks and operational standards in this rapidly growing industry.

## 2 Methods

### 2.1 Study Area

**FIGURE 1:**
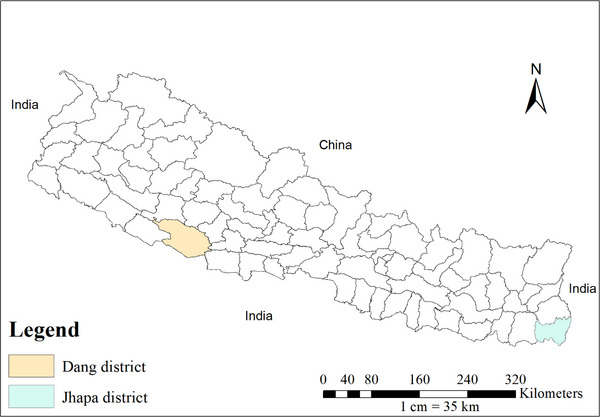
Map of Nepal showing Dang and Jhapa districts. The map was prepared using Arc GIS ver. 10.8, and shape file was obtained from Open Data Nepal (https://opendatanepal.com/dataset/new‐political‐and‐administrative‐boundaries‐shapefile‐of‐nepal).

The study was conducted in Dang and Jhapa districts of Nepal (Figure 1). These districts were selected purposively because of the high pig population and the importance of pig production as a key livelihood source for farmers in these districts. According to the MOALD (2022), with 92,161 and 74,000 pigs, Dang and Jhapa rank first and third, respectively, in pig population in Nepal.

Dang lies in Lumbini province bordering Pyuthan, Rolpa and Salyan in the north; Uttar Pradesh, India in the south; Argakhachi, Kapilvastu in the east; and Surkhet, Banke in the west. It is located at latitudes 27°36′ N to 28°1′ N and longitudes 82°2′ E to 82°5′ E. Dang has two sub‐metropolitan cities, one municipality and seven rural municipalities (Statistics Office, Dang 2023).

Jhapa lies in eastern Terai bordering Illam in the north, Morang in the west, Bihar, India in the south and West Bengal, India to the southeast and east. Geographically, it is 1606 km^2^ in area and lies at 87°39′ E to 88°12′ E longitude and 26°20′ N to 26°50′ N latitude. Jhapa has eight municipalities and seven rural municipalities (Statistics Office, Morang 2023).

### 2.2 Study Population, Study Design, Sample Size and Sampling Technique

According to the Veterinary Hospital and Livestock Service Expert Centre (VHLSEC) of the respective districts, there were a total of 123 pig farms in Dang and 127 in Jhapa. Out of the total 250 pig farms across the two districts, a smaple of 152 farms was selected using a finite population sample size calculation with a with a 95% confidence level and 5% margin of error.

### 2.3 Questionnaire Development

A researcher‐developed, expert‐validated and pre‐tested questionnaire was used to collect data. Initially, relevant literature was reviewed to understand biosecurity and associated issues in pig farms. Those literature included, but was not limited to Kouam et al. (2020); Kouam and Moussala (2018); Ogundijo et al. (2023); Ojabo and Enya (2020) and Silva et al. (2019). The questionnaire was then pre‐tested with 10 farmers to ensure that questions were clear, and they effectively captured the intended information, while also identifying any potential issues with survey flow, format or cultural sensitivity. Pre‐testing helped in enhancing the reliability and validity of data by addressing potential sources of error before administering the survey to a larger audience. The questionnaire was modified and finalized integrating the input received from pre‐test.

### 2.4 Data Collection Method

The data were collected through face‐to‐face interviews. The questionnaire was delivered to farm owners and, when owners were unavailable, the responsible person present at the farm was surveyed. We chose face‐to‐face interview as they have higher response rate and allow greater clarity (Bennink et al. 2013; Couper and Zhang 2016). The survey instrument was in English language and was placed on Kobo Toolbox. Two interviewers conversant with Nepali, Hindi and English languages visited the sampled farms and shared the questions in Nepali language (national language) to ease the understanding. Prior to asking questions, verbal consent was obtained from respondents, and they were informed of the objectives of the study, were assured of confidentiality of the data they would provide, and anonymity of their individual identities. Only those subjects who consented to voluntarily participate were interviewed. Before the start of the survey ethical clearance was obtained from Nepal Veterinary Council (Reference number 37/2080/81). The data were collected from November to December 2023. On average, it took about 30 min to interview a subject.

### 2.5 Data Analysis

The collected data were imported into IBM SPSS Version 23. The data were cleaned and coded. For every good biosecurity practice that subjects indicated they were adopting, a score of ‘1’ was given, and a score of ‘0’ was given for those who indicated they were not adopting such practice. Good biosecurity means implementing recommended practices that prevent the introduction and spread of diseases. A total biosecurity score was computed for each farm.

## 3 Results

### 3.1 Socio‐Demographics of Respondents

Out of the total 152 respondents interviewed, males and females accounted for 53.3% and 46.7%, respectively (Table 1). The majority of the respondents (69.1%) were of age between 36 and 60 years. The overwhelming majority of respondents were Aadibasi and Janajati (91.5%) and over two‐thirds of them (69.7%) followed Hinduism. About two‐thirds of them had completed the 10^th^ grade (63.6%). Only about one‐fifth of them (19.1%) reported pig farming as their primary occupation. In addition, 36.2% of the respondents had received training on pig production, and 11.2% had received training on biosecurity in pig farms.

**TABLE 1:** Respondents' demographics.

Respondents' demograraphics		Dang *n* = 84	Jhapa *n* = 68	Total *N* = 152
Variables	Categories			
Gender	Male	54.8%	51.5%	53.3%
	Female	45.2%	48.5%	46.7%
Age	Up to 35 years	31.0%	17.6%	25.0%
	36 to 60 years	66.7%	72.1%	69.1%
	61 and above	2.3%	10.3%	5.9%
Ethnicity	Aadibasi and Janajati	86.9%	97.1%	91.5%
	Brahmin	1.2%	1.5%	1.3%
	Chhetri	11.9%	1.4%	7.2%
Religion	Hinduism	97.6%	35.3%	69.7%
	Kirat	0.0%	30.9%	13.8%
	Buddhism	0.0%	17.6%	7.9%
	Christianity	1.2%	7.4%	3.9%
	Others	1.2%	8.8%	4.7%
Education	No formal education	30.1%	16.2%	23.8%
	School level education (10 years of education)	62.7%	64.7%	63.6%
	College and university level (greater than 10 years of education)	7.2%	19.1%	12.6%
Pig farming as a primary occupation	No	84.5%	76.5%	80.9%
	Yes	15.5%	23.5%	19.1%
Ever attended pig production training	No	59.5%	69.1%	63.8%
	Yes	40.5%	30.9%	36.2%
Ever attended biosecurity training	No	84.5%	94.1%	88.8%
	Yes	15.5%	5.9%	11.2%

### 3.2 Farm Characteristics and Management

The majority of the land where the pig farms were housed was self‐owned (71.1%) (Table 2). More than half of the farms surveyed follow a semi‐intensive (56.6%) farming practice. The majority of the farmers (69.1%) used swill feeding to feed their pigs. Most farms were managed exclusively by owners themselves (94.7%), with only a few farms (3.3%) being managed by hired farm workers. Only a few farms (4.6%) maintained farm records. About half of the farms (53.9%) regularly vaccinated their pigs against major infectious diseases. Of those that vaccinated their pigs, 24.3% vaccinated against FMD only, and 21.7% vaccinate against FMD and CSF. A few farmers (15.1%) reported any disease outbreak in their farms to the relevant authorities. Only a small number of farmers (5.9%) regularly visited forest areas, while 42.1% never visited forest areas.

**TABLE 2:** Farm characteristics and management practices in farms.

Management practice		Dang	Jhapa	Total
Variables	Categories	*n* = 84	*n* = 68	*N* = 152
Ownership of farm land	Rented or leased	36.9%	11.8%	25.7%
	Self‐owned	59.5%	85.3%	71.1%
	Self and rented both	3.6%	2.9%	3.2%
Management practice type	Extensive^a^	8.3%	0.0%	4.6%
	Semi‐intensive^b^	64.3%	47.1%	56.6%
	Intensive^c^	27.4%	52.9%	38.8%
Swill feeding	No	25.0%	38.2%	30.9%
	Yes	75.0%	61.8%	69.1%
Farms looked by	Self (owner)	95.2%	94.2%	94.7%
	Workers	3.6%	2.9%	3.3%
	Both	1.2%	2.9%	2.0%
Maintaining farm record book	No	92.9%	98.5%	95.4%
	Yes	7.1%	1.5%	4.6%
Vaccination in pigs	No	48.8%	42.6%	46.1%
	Yes	51.2%	57.4%	53.9%
Vaccination type	Against CSF only	14.3%	0.0%	7.9%
	Against FMD only	10.7%	41.2%	24.3%
	CSF and FMD vaccinated	26.2%	16.2%	21.7%
Reporting the outbreak(s)	No	82.1%	88.2%	84.9%
	Yes	17.9%	11.8%	15.1%
Visiting forest areas	Never	39.3%	45.6%	42.1%
	Often	32.1%	39.7%	35.5%
	On a regular basis	2.4%	10.3%	5.9%
	Seldom	26.2%	4.4%	16.5 %

^a^ Animals of relatively small number are permanently penned and fed on agriculture by products and kitchen waste.

^b^ Animals are permanently penned in piggeries with a rough cast floor, fed on kitchen waste, agricultural by‐products and often industrial feed.

^c^ Animals are improved breeds, indoors, in high number, the piggeries are a modern building, feedstuff is exclusively industrial.

### 3.3 Biosecurity—Isolation

Table 3 shows data on the isolation component of biosecurity practices. Farms which were located at a distance equal to or greater than 501 m from motorable roads, forest areas and neighbouring livestock operations were 1.3% (*n* = 2), 70.4% (*n* = 107) and 23% (*n* = 35), respectively. Only 15.8% (*n* = 24) of the farms had a fence. About half of the farms (53.9%) practiced quarantine of newly introduced pigs. A majority of the farms (86.2%) had provisions for segregating animals by age and sex. In addition, 57.2% (*n* = 87) of the farms reared animals other than pigs on the same premises. A few farms (14.5%) followed proper segregation of sick pigs in a sick pen.

**TABLE 3:** Biosecurity—isolation component in study area.

S.N.	Isolation component		Dang *n* = 84	Jhapa *n* = 68	Total *N* = 152
	Variable	Categories			
1	Distance from motorable roads	≤ 500 m	97.6%	100.0%	98.7%
		≥ 501 m^a^	2.4%	0.0%	1.3%
2	Distance from nearest forest area	≤ 500 m	27.4%	32.4%	29.6%
		≥ 501 m^a^	72.6%	67.6%	70.4%
3	Distance from nearest livestock operation	≤ 500 m	72.6%	82.4%	77.0%
		≥ 501 m^a^	27.4%	17.6%	23.0%
4	Fencing of farm	No	76.2%	94.1%	84.2%
		Yes^a^	23.8%	5.9%	15.8%
5	Quarantine of newly introduced pigs	No	48.8%	42.6%	46.1%
		Yes^a^	51.2%	57.4%	53.9%
6	Age and sex related differentiation of pigs	Both age and sex related differentiation is done^a^	84.5%	88.2%	86.2%
		Only age‐related differentiation is done	6.0%	2.9%	4.6%
		Only sex related differentiation is done	9.5%	8.9%	9.2%
7	Raising other domestic animals in the farm	No^a^	42.9%	42.6%	42.8%
		Yes	57.1%	57.4%	57.2%
8	Proper segregation of sick pigs in sick pen	No	79.8%	92.6%	85.5%
		Yes^a^	20.2%	7.4%	14.5%

^a^ Good biosecurity practices.

### 3.4 Biosecurity—Traffic Control

Table 4 shows the data on traffic control component of biosecurity. None of the farms followed linear flow principles or the practice of assigning a single person to manage a single shed. In addition, 57.9% (*n* = 88) farms didn't use farm equipment for other purposes, while 8.6% (*n* = 13) of farms exchanged farm equipment with other farms. The majority of farms (77.6%) allowed visitors into their farm. More than half of the farms (56.6%) used boars from others' farms for breeding, and 33.6% (*n* = 51) provided their own farm boars for breeding in other farms. In addition, 36.8% of farms kept pet animals, while farms with a single entrance point and signs depicting a restriction zone were 13.2% (*n* = 20) and 2.0% (*n *= 3), respectively. None of the farms followed all‐in‐all‐out practice.

**TABLE 4:** Biosecurity—traffic control component in study area.

	Traffic control component				
S.N.	Variables	Categories	Dang *n* = 84	Jhapa *n* = 68	Total *N* = 152
1	Linear flow principle	No	100.0%	100.0%	100.0%
		Yes^a^	0.0%	0.0%	0.0%
2	Single shed managed by single person	No	100.0%	100.0%	100.0%
		Yes^a^	0.0%	0.0%	0.0%
3	Farm equipment not used for other purposes	No	46.4%	36.8%	42.1%
		Yes^a^	53.6%	63.2%	57.9%
4	Exchange of farm equipment with other farms	No^a^	85.7%	98.5%	91.4%
		Yes	14.3%	1.5%	8.6%
5	Allowing visitors to enter farm compartment	No^a^	17.9%	27.9%	22.4%
		Yes	82.1%	72.1%	77.6%
6	Use of other farms boar to breed sow	No^a^	45.2%	41.2%	43.4%
		Yes	54.8%	58.8%	56.6%
7	Use of own farm boar to breed other farms sow	No^a^	63.1%	70.6%	66.4%
		Yes	36.9%	29.4%	33.6%
8	Pet animals	No^a^	63.1%	63.2%	63.2%
		Yes	36.9%	36.8%	36.8%
9	Signs depicting restriction zone	No	97.6%	98.5%	98.0%
		Yes^a^	2.4%	1.5%	2.0%
10	Single entrance point	No	79.8%	95.6%	86.8%
		Yes^a^	20.2%	4.4%	13.2%
11	All‐in‐all out practice	No	100.0%	100.0%	100.0%
		Yes^a^	0.0%	0.0%	0.0%

^a^ Good biosecurity practices.

### 3.5 Biosecurity—Sanitation

Only 2.6% (*n* = 4) of the farms disinfected vehicles entering the premises, and only 5.3% (*n* = 8) had a functional footbath (Table 5). A small proportion (6.6%) used clean coveralls and boots while entering the farm. Although 67.1% (*n* = 102) of farmers cleaned their farm compartments and premises daily, only 9.2% (*n* = 14) used disinfectants for cleaning piggeries. Only 3.3% (*n* = 5) of the farms regularly disinfected drinking water. A well‐managed drainage was present in only 9.9% (*n* = 15) of the farms. In addition, only 4.6% (*n* = 7) of the farms took measures to prevent feedstuffs from contamination by rodents. Dead pigs were generally disposed through burials, burning (98.3%) or occasionally used as feedstuff for other animals (1.7%). A sanitary lock system was absent on all the farms covered under this study. Only 2.6% (*n* = 4) of farms had a designated gowning zone. The surveyed farms did not strictly follow pest control programmes; only 5.3% (*n* = 8) implemented rodent control, 2.6% (*n* = 4) implemented flies' control, and 2.0% (*n* = 3) implemented birds' control programmes.

**TABLE 5:** Biosecurity—sanitation component in study area.

S.N.	Sanitation component		Dang *n* = 84	Jhapa *n* = 68	Total *N* = 152
	Variable	Categories			
1	Disinfect vehicles entering farm	No	95.2%	100.0%	97.4%
		Yes^a^	4.8%	0.0%	2.6%
2	Has functional footbath	No	91.7%	98.5%	94.7%
		Yes^a^	8.3%	1.5%	5.3%
3	Use of clean coveralls and boots	No	91.7%	95.6%	93.4%
		Yes^a^	8.3%	4.4%	6.6%
4	Clean piggeries daily	No	34.5%	30.9%	32.9%
		Yes^a^	65.5%	69.1%	67.1%
5	Use of disinfectant to clean the piggeries	No	90.5%	91.2%	90.8%
		Yes^a^	9.5%	8.8%	9.2%
6	Regularly disinfecting drinking water	No	96.4%	97.1%	96.7%
		Yes^a^	3.6%	2.9%	3.3%
7	Has a well‐managed drainage system	No	90.5%	89.7%	90.1%
		Yes^a^	9.5%	10.3%	9.9%
8	Prevent feedstuff from rodents	No	92.9%	98.5%	95.4%
		Yes^a^	7.1%	1.5%	4.6%
9	Disposal of dead pigs	Buried or burnt^a^	98.8%	100.0%	98.3%
		Fed to other animals	1.2%	0.0%	1.7%
		Consumed for meat	0.0%	0.0%	0.0%
10	Presence of sanitary lock	No	100.0%	100.0%	100.0%
		Yes^a^	0.0%	0.0%	0.0%
11	Gowning area	No	95.2%	100.0%	97.4%
		Yes^a^	4.8%	0.0%	2.6%
12	Rodent control programme	No	94.0%	95.6%	94.7%
		Yes^a^	6.0%	4.4%	5.3%
13	Flies control programme	No	97.6%	97.1%	97.4%
		Yes^a^	2.4%	2.9%	2.6%
14	Bird control programme	No	97.6%	98.5%	98.0%
		Yes^a^	2.4%	1.5%	2.0%

^a^ Good biosecurity practices.

### 3.6 Overall Biosecurity Score

**FIGURE 2:**
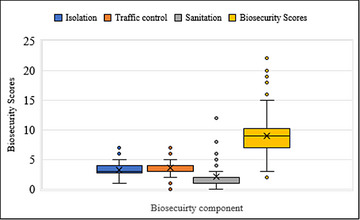
Box and whisker plot of biosecurity scores of pig farms in Dang and Jhapa of Nepal.

The farm biosecurity scores were calculated across three components: isolation, traffic control and sanitation. Each farm could get a maximum of 33 points comprising 8 for isolation, 11 for traffic control and 14 for sanitation. The average scores for isolation, traffic control and sanitation were 3.3 ± 1.2, 3.6 ± 1.4 and 2.2 ± 1.5, respectively (Figure 2). Overall, farms achieved an average biosecurity score of 9.1 ± 3.0 (Figure 2), indicating that farms under this study do not have good biosecurity.

## 4 Discussion

This study examined management and biosecurity practices of pig farms in Dang and Jhapa of Nepal, the two pig production pockets in the country. The findings indicate that primarily Aadibasi and Janajati communities raised pigs which is consistent with common trends observed across Nepal. This pattern reflects a long standing cultural norms influencing pig production and consumption. In Dang, the majority of the pig farmers were Hindus, whereas the population in Jhapa consisted of Hindus, Kirats and Buddhists. About one‐fourth of the pig farmers had no formal education. Only a small proportion considered pig farming as their primary occupation. Many farmers did not receive any training in pig rearing and biosecurity. It is essential for local authorities to maintain up‐to‐date records of businesses operating within their service areas. This will be helpful to authorities to check compliance with local regulations, conduct timely monitoring, and respond promptly when intervention is required, particularly during incidences such as disease outbreaks.

The majority of farms followed a semi‐intensive production system. They commonly fed swill to their pigs. Swill feeding of pigs is widely practiced globally, particularly among smallholders in Belgium (Ribbens et al. 2008), in Cameroon (Kouam et al. 2020), in Kenya (Kagira et al. 2010), in Philippines (Andiko and Pena 2019), and India (Singh et al.^2023^). It could be because swill is easily available and is cheaper than other feed sources. However, swill can act as a source of deadly pathogens (Hernández‐Jover et al. 2016). Swill, its sources, and its use are not regulated in Nepal, creating substantial biosecurity risks.

Vaccination of pigs against major contagious diseases can prevent the respective disease outbreaks and there are several such vaccinations recommended for pigs (Blome et al. 2017; Cox and Barnett 2009). Our study shows that the majority of farmers didn't consider vaccinating their pigs as a top priority or necessity. Only half of the farms regularly vaccinated their pigs against FMD or CSF or both. Some vaccines were provided free of charge to farmers by VHLSEC, but coverage gaps may result from limited technician outreach and farmers' lack of awareness regarding routine vaccination.

Around one‐fourth of farms were at proximity to the forests. Farms near forests have high chance of contact between domestic pigs and wild boars, which increases the risk of pathogens spill over from wild boars to domestic pigs (Guinat et al. 2016; Thakur et al. 2024). In addition, 77% of the pig farms under this study were found to be within a distance of 500m from another livestock operation. This poses a risk of transmission of diseases from one farm to another either through aerosol routes (Brown et al. 2022) or by direct contact where biosecurity measures are not followed. Most pig farms reported not having fences on their premises which is similar with the findings of Kouam et al. (2020); Kadja et al. (2021) and Singh et al. (2023). High initial investment required for fencing might have prevented farmers from investing in fencing their farm premises. Fencing around farms and putting signs to regulate entry of people and goods in the farm premises greatly help to control intrusion of visitors and feral animals. The benefit of fencing outweighs the cost incurred during fencing. Similarly, quarantine acts as a reliable measure of preventing disease entry into the farm and it is critically important in livestock and poultry farms (Food and Agriculture Organization 2010). Only half of the farms under study followed quarantine which indicates that farmers seem to be ignorant of it. In the same line, another finding shows that 57.2% of the pig farmers raised other livestock species along with pigs on the farm premises indicating that there was high chances of cross‐species transmission of diseases (Miller et al. 2017). Furthermore, if the same personnel work in two different sheds with two livestock species there is also likelihood of cross‐transmission of pathogens across species and sheds through those workers. To prevent the spread of diseases within the farm, the farms need to practice proper isolation of sick animals.

None of the farms under this study followed linear flow principle of management. The linear flow principle is the movement of caretakers and visitors in a single direction. This approach helps maintain a clear separation between clean areas and dirty areas and limits pathogens spillover from between these areas, thereby minimimizing the risk of disease spread throughout the farm. On another note, 8.6% of the farms exchanged farm equipment with other farms. While exchanging equipment, if no hygiene and sanitation is taken care of, which is very likely in small farms with little or no knowledge of biosecurity, pathogens can enter the farm (Otake et al. 2002). Not only the equipment or innate objects, but the majority of the farms revealed that they shared breeding boars between farms which should be discouraged from the biosecurity perspective. Breeding animals may appear apparently healthy yet carry infectious agents, which can prove costly. Contact with infected animals, through aerosol and contaminated semen are some possible disease transmission routes, for example, ASF (Friedrichs et al. 2022), CSF (De Smit et al. 1999), FMD (Paton et al. 2018), Aujeszky's Disease (Bo and Li 2022) and Brucellosis (Olsen and Tatum 2016). It is common to keep pet animals in farms for the security reason, but they add further risk due to their foraging behaviour (Mor et al. 2016). Here, the all‐in‐all‐out method of livestock rearing is worth discussing. This refers to the complete emptying (of animals) of a room, sheds or building and litters and other things inside the sheds such as waterers or other tools, etc. which could harbour pathogens and their cleaning and disinfection (without the animals inside) before introducing a new batch. This management practice avoids contact of animals of different ages and batchescutting offthe infection cycles of the pathogens. This process is very effective in maintaining farm biosecurity and preventing possible disease outbreaks. As most farms in Nepal are small or medium scale, it was not surprising knowing that none of the farms followed all‐in‐all‐out system. Similar was the finding of Singh et al. (2023) in India, where they reported only 1.8% farms followed all‐in‐all‐out protocol. This low adoption of all‐in‐all‐out protocol ap could be due to limited space, financial constraints and traditional management practices among farmers of Nepal.

Pig farms could be infested by pests like rodents, birds, flies, tick and fleas which can act as mechanical vector of numerous pathogens (Alarcón et al. 2021). The farms under this study lacked structured pest control programmes. Maintaining sanitary condition is an important aspect of any farm management practices. The farms in this study performed below average on many indicators of sanitation component which show huge gap in pig production in Nepal. Previous studies have found that fomites (Otake et al. 2002), and transport vehicles (Dee et al. 2004; Rajao et al. 2019) are potential source for the introduction of diseases in the farm. Regularly cleaning and disinfection of piggeries using effective disinfectants are essential to kill potential pathogens present on the farm (Divyalakshmi et al. 2016). Only 9.9% of the farms have adopted a well‐managed drainage system. The drainage system plays a vital role in maintaining sanitary condition in farm premises. Improper drainage can harbor pathogens and act as a source of diseases in the farm as it has direct access to flies and rodents. None of the farms in our study followed sanitary lock system. The sanitary lock system is a practice of separating dirty areas from clean area where workers or visitors need to go through number of compartments to change their clothes and clean their bodies to prevent entry of risk into the farm compartment.

Overall biosecurity performance in pig farms under this study was low, with a mean score of 9.1 ± 3.0 out of maximum possible score of 33. Biosecurity is essential and integral to farms to ensures good herd health and provide financial stability. Though the initial investment in biosecurity is higher, but in the long run it helps reduce potential cost incurred due to disease outbreak (Fasina et al. 2012). Farmers not accepting pig farming as their primary profession and th majority of farms being small‐scale, carried mainly for sustenance, likely contribute to poor biosecurity adoption.

Aligning with findings of this study, Acharya and Wilson (2020) note that conventional pig farming which is characterized by weak management practices is dominant in Nepal. This is supported by the study done by Plut et al. (2023) in Slovenia, where large‐scale farms demonstrate significantly stricter adherence to biosecurity protocols compared to small‐scale holdings. The lack of knowledge about biosecurity practices among pig farmers may have also caused reduction in the adoption of proper biosecurity practices. The domestication and illegal hunting of wild pigs also exacerbate the situation (Shrestha et al. 2014), as wild pigs are the reservoir of various deadly pathogens including ASF and CSF (Guinat et al. 2016). It is therefore essential for farmers to be adequately informed of and educated about these diseases, as well as prevention and management strategies (Bremang et al. 2022).

Improving biosecurity in Nepal's pig farms requires addressing multiple interconnected constraints, including limited awareness, financial limitations, inadequate infrastructures and insufficient institutional support. Most smallholder farmers have not received formal training on pig production and biosecurity, they lack the resources to invest in fencing, and drainage or quarantine facilities; and therefore they often manage pigs as a secondary source of income. While simple measures like restricting visitors, isolating sick pigs or cleaning with disinfectants can be adopted through better education and awareness, more resource‐intensive improvements require coordinated assistance. Sustainable progress demands shared responsibility: farmers must take initiative in applying feasible, low‐cost practices, while government agencies, local authorities and development partners should provide targeted training, financial incentives and clear regulatory guidance. A coordinated approach that links knowledge, resources and governance is essential to make biosecurity practices practical, equitable and effective across Nepal's diverse pig farming systems.

## 5 Limitation of the Study

The major limitation of the study is the small sample size. The snowball sampling very likely introduces biases such as selection bias, researcher bias, social desirability bias and network bias in research. We didn't use weightage system assuming every practice has equal impact on biosecurity. We used cross‐sectional study, which might have overlooked the seasonal or other causal effects.

## 6 Conclusion and Recommendation

This study provides critical insights into the management and biosecurity practices of pig farms in Dang and Jhapa, Nepal, revealing significant setbacks that increases the vulnerability of farms to disease outbreaks. These findings underscore the need for immediate interventions targeting capacity building of, knowledge dissemination among and adoption of evidence‐based biosecurity practices by pig farmers. Future research can be on to investigate scalable and cost‐effective biosecurity solutions tailored to small‐scale and semi‐intensive pig farming systems in Nepal and other countries with similar farming systems. From a technology transfer perspective, government and industry stakeholders should prioritize the development and dissemination of affordable technologies and farmer‐friendly tools. Moreover, introductionof insurance schemes and financial incentives abiosecurity technologies and reduce disease‐related economic losses.

## Author Contributions

**Sachin Shrestha**: conceptualization, investigation, writing – original draft, methodology, validation, visualization, writing – review and editing, software, formal analysis, project administration, resources, supervision, data curation. **Alok Dhakal**: conceptualization, writing – original draft, methodology, validation, visualization, writing – review and editing, software, formal analysis, project administration, resources, supervision. **Ramjee Ghimire**: conceptualization, writing – original draft, methodology, validation, visualization, writing – review and editing, software, formal analysis, project administration, resources, supervision, data curation. **Min Bahadur Oli**: investigation, writing – original draft, methodology, writing – review and editing, software, formal analysis, data curation. **Rakesh Kumar Yadav**: conceptualization, writing – original draft, formal analysis, data curation, project administration, supervision, resources. **Nishant Shah**: writing – original draft, project administration, supervision, resources. All authors reviewed the results and approved the final version of the manuscript.

## Funding

The project was funded by Association of Nepalese Agricultural Professionals of Americas (NAPA) under the NAPA Research Mini‐Grant 2022 program.

## Ethics Statement

Nepal Veterinary Council (NVC) reviewed and approved the research (NVC: 37/2080/81). Verbal consent was obtained from the research participants for their voluntary participation in the study prior to collecting the data.

## Consent

The participant gave verbal consent to participate in the study.

## Conflicts of Interest

The authors declare no conflicts of interest.

## Data Availability

Study data and related materials are available upon reasonable request from the corresponding author.
